# Comparing the Effects of Mineral Trioxide Aggregate and Calcium Enriched Mixture on Neuronal Cells Using an Electrophysiological Approach

**Published:** 2012-06-01

**Authors:** Fatemeh Abbasipour, Vahid Akheshteh, Ali Rastqar, Habib Khalilkhani, Saeed Asgary, Mahyar Janahmadi

**Affiliations:** 1. Neuroscience Research Centre and Department of Physiology, Medical School, Shahid Beheshti University of Medical Sciences, Tehran, Iran; 2. Department of Periodontics, Dental School, Hamadan University of Medical Sciences, Hamadan, Iran; 3. Dental Research Center, Shahid Beheshti University of Medical Sciences, Tehran, Iran; 4. Iranian Centre for Endodontic Research, Dental School, Shahid Beheshti University of Medical Sciences, Tehran, Iran

**Keywords:** Action Potential, Calcium Enriched Mixture, CEM Cement, Helix Aspersa, Intracellular Recording, MTA, Neuronal, White Mineral Trioxide Aggregate

## Abstract

**Introduction:**

The main goal of this ex vivo study was to assess and compare the cellular and electrophysiological effects of two dental biomaterials, white mineral trioxide aggregate (WMTA) and calcium enriched mixture (CEM) cement, on neuronal cell excitability and electrical properties.

**Materials and Methods:**

A conventional intracellular current clamp technique was used to study the cellular effects of WMTA and CEM on the excitability, firing and the shape of action potential of neuronal soma membrane of F_1_ nerve cells. The dental biomaterials were prepared according to the manufacturers' directions and were applied to the bathing media and 0.05 mL of total mixture of each dental material at a distance of 3 mm from the cells.

**Results:**

Findings indicated that exposure to both dental biomaterials shifted the irregular high frequency firing type observed in control conditions to a more regular low frequency firing pattern. Neuronal exposure to WMTA, but not CEM, significantly hyperpolarized the cell resting membrane potential. Both treatments significantly influenced the duration and the amplitude of action potentials. Extracellular application of either CEM or WMTA caused a significant increase in the after hyperpolarization (AHP) amplitude and AHP area, but the potentiating effect of WMTA was more effective than CEM.

**Conclusion:**

Treatment with WMTA or CEM resulted in a profound alteration in the firing behaviour of F_1_ cells and changed the AP characteristics. Both dental biomaterials reduced the neuronal activity possibly through enhancement of K^+^ outward current. This may possibly explain the positive mechanisms of these biomaterials in regenerative endodontics, though further research is needed for such a conclusion.

## Introduction

Biocompatibility and non-toxicity are two important properties required for ideal biomaterials which are used for pulp capping and root-end fillings [1]. Substances and materials used in these endodontic therapies may come into close contact with living biological tissue; therefore, cellular responses to these materials are of particular interest.

White mineral trioxide aggregate (WMTA) and calcium enriched mixture (CEM) cement are two dental biomaterials with several clinical applications, including pulp capping, pulpotomy, root-end filling and perforation repair [2][3][4][5][6]. Preservation and maintenance of pulpal vitality is very important in endodontics. Therefore, bioactive materials that can stimulate the cellular repair phenomenon and promote the formation of dentin bridge would be highly beneficial. MTA, largely because of its small particle size, sealing ability, alkaline pH and slow release of Ca^2+^ has been shown to be an effective pulp capping material [7][8][9]. On the other hand, CEM, which has a different chemical composition than MTA [10], exhibits comparable biological outcomes with MTA when used as a root-end filling [11] or pulp capping material [4][12][13][14][15]. The ability of both cements to induce dentinogenesis has also been reported [5][16][17]. Despite intensive research on physical/chemical/clinical/biological properties of these biomaterials [3][4][5][6][7][8][9][10][11][12][13][14][15][16][18][19][20][21][22][23][24], the cellular effect of either MTA or CEM is not well understood, because there is lack of in vivo and/or in vitro studies that provide detailed information regarding cellular events such as exposure to excitable tissue, namely, neuronal cells. In excitable cells the generation of an action potential is a complex process that involves the temporal opening and closing of voltage-dependent ion channels within the cell membrane. Changes of these ion channels by the application of drugs and biochemical including dental materials can alter the configuration of action potential. Thus, action potential shape analysis could be a valuable tool for the measurement of drug effects based on their cellular mechanism of action.

The aim of the present ex vivo study was to assess and compare the electrophysiological effects of WMTA and CEM on F_1_ neuronal excitability in Helix aspersa using intracellular recording techniques.

## Materials and Methods

### Intracellular Recording

Experiments were performed on F_1_ neurones from the right parietal lobe of suboesophageal ganglia of the Iranian garden snail, Helix aspersa. Animal dissection was performed as previously described [25][26]. Briefly, the ganglionic mass was dissected out and pinned by the nerves and edges of the connective tissue into a Sylgard (Dow Corning Midland, MI, USA)-grounded recording chamber with a total volume of 1 mL, and the overlying layers of connective tissue were mechanically torn using fine forcipes in order to expose F_1_ neuron cell bodies. All intracellular recordings were performed at room temperature (21-24°C) and in snail Ringer containing NaCl, 84 mM; CaCl2, 10 mM; KCl, 4 mM; MgCl2, 5 mM; glucose, 10 mM; HEPES, 5 mM; pH adjusted to 7.4 with TRISMA-base.

**Figure 1:**
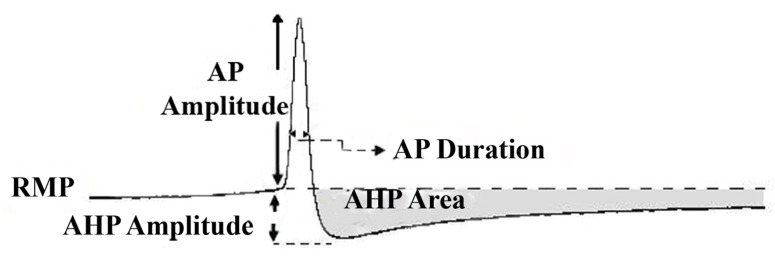
Measurements of action potential properties; RMP, resting membrane potential; AP Amplitude, Action potential and duration; AHP, after hyperpolarizations amplitude and area under the AHP

All research and animal care procedures were performed according to the protocols approved by Shahid Beheshti University of Medical Sciences ethical committee for animal research.Conventional intracellular recordings under current clamp condition were conducted using Axoclamp 2B amplifier (Axon Instrument, Foster City, CA, USA). To study the behavior of the neuronal membrane potential, or to understand how a neuron can be excited and inhibited, a current clamp technique is used in which the voltage difference across the cellular membrane is measured while injecting a constant current into the cell. The electrical responses of neuronal cells were recorded with microelectrodes (Clark Instrument, UK), which were pulled with a vertical puller (PC-10, Narishige, Japan) and had resistances ranging from 3.5-5 milliohm when filled with 3MKCl. Sampled data were digitized using an A/D converter (AD Instrument, Australia) and stored on an IBM computer with Chart software for offline analysis. The following quantitative parameters of action potential (AP) were measured using either Chart 6 software (AD Instrument, Australia) or Minianalysis (Synaptosoft Inc., Decatur, GA): The resting membrane potential (RMP), the amplitude of AP, which was defined as the change in voltage from the RMP to the peak of the AP. The duration of the AP and AHP amplitude were measured at half amplitude and from the RMP to the maximum negativity after an AP, respectively. The area under the curve for AHP (AHP area, mVs) of the AP was also measured (Figure 1) and to quantify the neuronal firing regularity, coefficient of variation (CV) of the interspike interval (ISI) was calculated as standard deviation of ISI /mean ISI.

### Treatment

White ProRoot MTA (Dentsply, Tulsa Dental, Tulsa, OK, USA) was prepared according to the manufacture’s direction: as a mixture of powder (20 mg) and normal Ringer (0.2 mL) in a slurry form [27]. A recently introduced biomaterial called CEM cement (BioniqueDent, Tehran, Iran) was also prepared as a creamy mixture with phosphate solution (i.e. CEM vehicle; 20 mg/0.2 mL normal Ringer). Then, 0.05 mL of the mixtures was separately added to the recording chamber containing extracellular solution.

Experiments were conducted on four groups of snail neurons: WMTA treated (n=11), CEM-treated (n=17) and two separate control groups for each experimental group (n=18, in each group). From each snail, one F_1_ neuron was recorded only once in control conditions and after applying consequent treatment. The two experimental groups were independent of each other. After 15 min of control recording, WMTA or CEM was independently added to the extracellular media close to the right parietal ganglion. The recording was continued for 80 min. The maximum cellular effects which occurred between 25 and 35 min after application of either WMTA or CEM were used for analysis.

The effect of both dental materials was irreversible upon wash-out for 20 min (data not shown). There were also no significant differences between the two control groups (Student t-test, P>0.1) in any of the electrophysiological parameters; therefore, the data were pooled for remaining analysis and presented as the control group.

Next, a series of experiments were conducted to verify the ability of CEM and WMTA to release Ca^2+^, and, thereby, to affect the amplitude of the AHP. To address this issue, the concentration of Ca^2+^ in the extracellular Ringer solution was reduced to half (i.e. reduced from 10 mM to 5 mM).

### Statistical analysis

Numerical results are given as mean ± SEM, with n being the number of cells on which the measurement was performed. Significant differences between the groups were evaluated using a Student’s t-test or one-way ANOVA and P<0.05 was considered to be significant.

## Results

**Figure 2:**
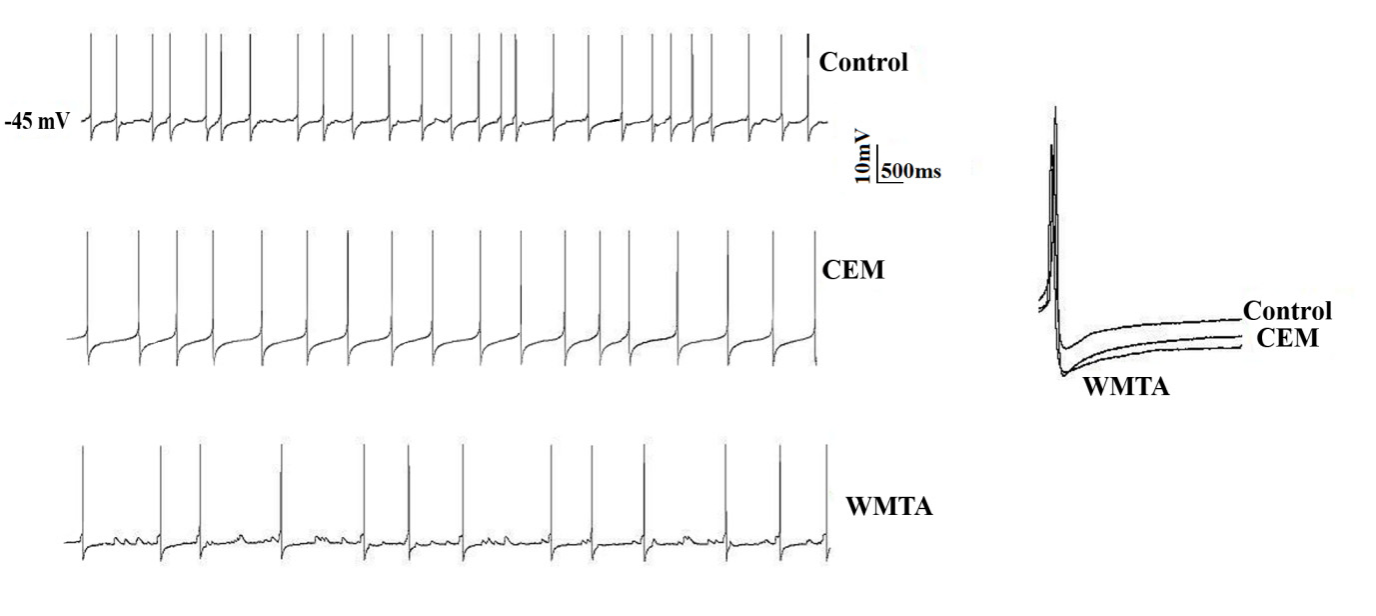
Effect of WMTA and CEM on firing pattern of F_1_ neurons; Intracellular recordings from F_1_ neurons show firing pattern in control condition (upper panel) and after extracellular application of CEM (middle panel) and WMTA (lower panel)

**Figure 3:**
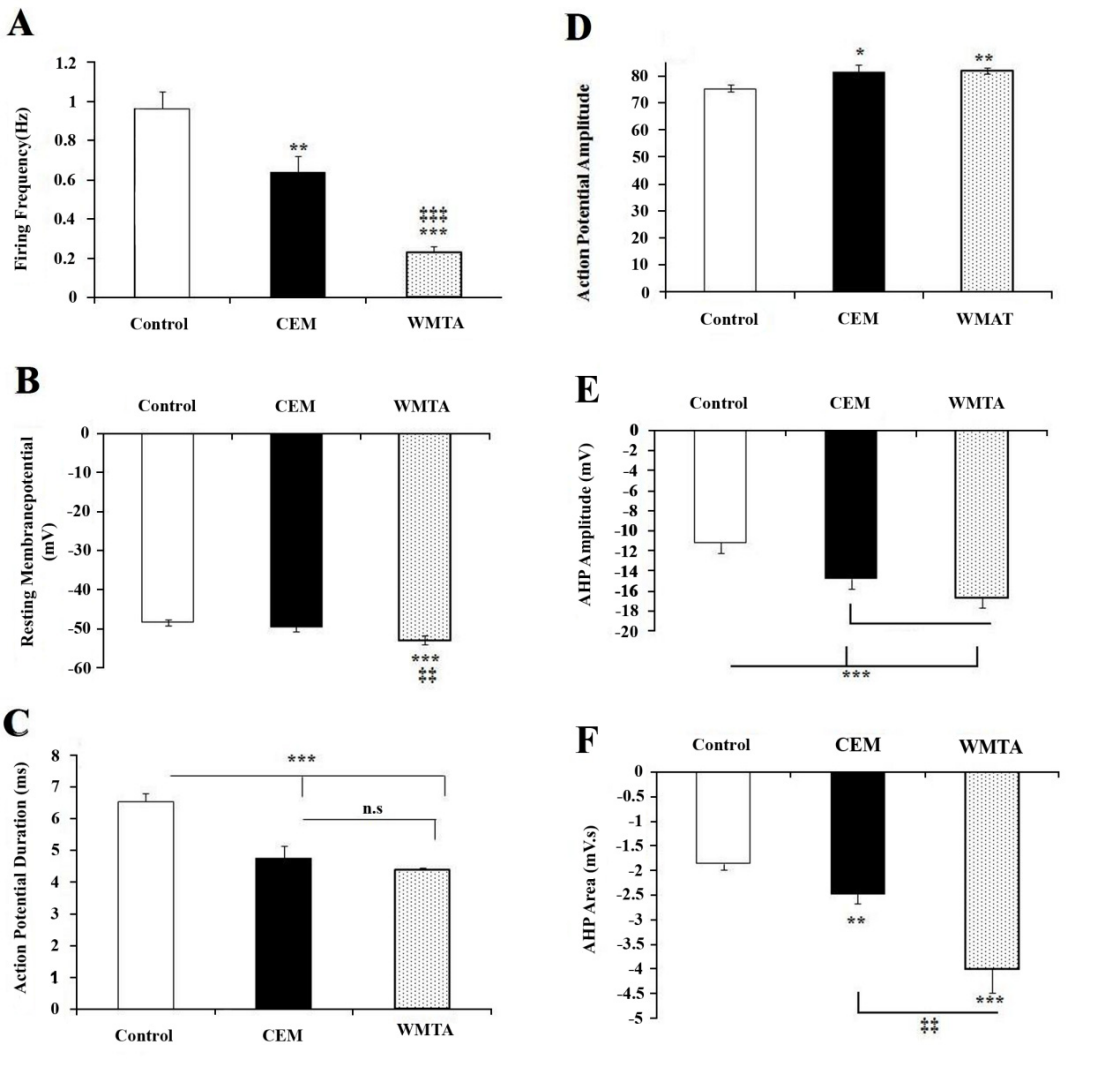
Effects of bath applications of CEM and MTA on action potential characteristics. Average data (mean ± SEM) showing the effect of CEM and WMTA on firing frequency; A) resting membrane potential; B) action potential duration; C) action potential amplitude; D) after hyperpolarizations (AHP) amplitude; E) and AHP area; F) One-way ANOVA was used to analyze the data. *,**,*** = significantly different (P<0.05, P<0.01, P<0.001, respectively) from the control; ‡‡ = significant difference (P<0.01) between CEM and WMTA

Intracellular recordings were obtained from a total of 64 F_1_ neurons in normal Ringer. Of these, 36 were from control, 11 from WMTA treated group, 17 from CEM treated group. These cells in control condition were spontaneously active and exhibited an irregular tonic firing pattern (Figure 2) with a mean frequency of 0.96±0.08 Hz (Figure 3A), as evidenced by a coefficient of variation of 0.53. They had a mean resting membrane potential of -48.36±0.67 mV (Figure 3B). The duration and the amplitude of APs were 6.54±0.25 ms and 75.33±1.6 mV, respectively (Figure 3C and Figure 3D). The AHP amplitude and the AHP area were -11.17±0.66 mV and -1.87 ± 0.12mV.s, respectively (Figure 3E and Figure 3F).

**Figure 4:**
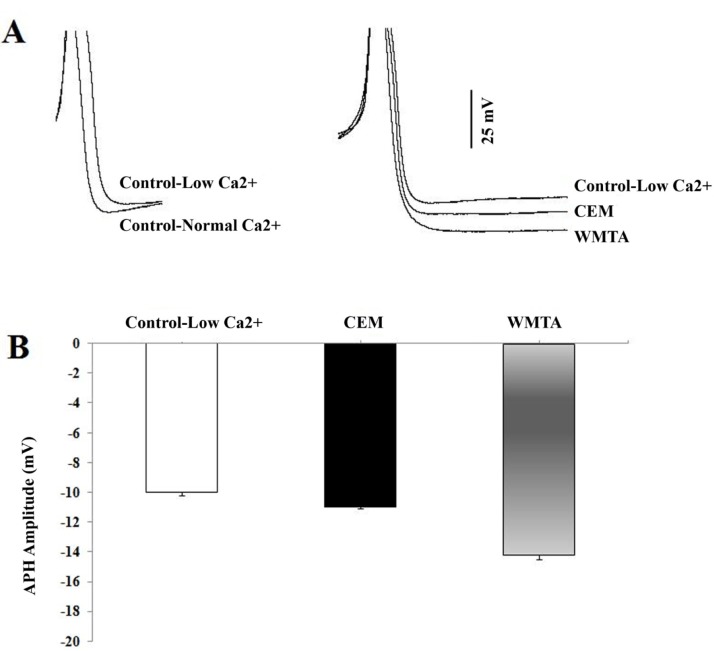
Effect of CEM and WMTA on the AHP amplitude recorded in low Ca^2+^ Ringer solution; A) Superimposed action potentials (truncated) recorded in normal Ringer and in low Ca^2+^ Ringer solution (left panel) and in the presence of CEM and WMTA application (right panel) into the bating solution containing low Ca^2+^ concentration; B) Bar graph summarising the effects of CEM and WMTA on the AHP amplitude measured in low Ca^2+^ concentration Ringer)

### Treatment with WMTA and CEM altered profoundly the electrophysiological characteri-stics of F_1_ neurons

To determine whether WMTA or CEM influences the neuronal excitability, electrophysiological characteristics of neuronal APs were compared to control recordings. Following extracellular application of either CEM or WMTA, F_1_ cells displayed a more regular firing activity (Figure 2) as evidenced by smaller CV (i.e. 0.361 and 0.362, respectively). This was associated with a significantly lowered firing frequency (0.63±0.08 Hz and 0.23±0.02 Hz, respectively). There was, however, a significant difference between CEM and MTA treated neurons in their firing frequency (Figure 3A). CEM treatment did not affect the RMP (-49.78±0.69 mV), but WMTA shifted the RMP to more negative voltages (-53.07±1.2 mV) compared to their counterparts in normal (P<0.001) or in CEM treated (P<0.01) cells Figure 3B. Both treatments resulted in a statistically similar, but significant differences in AP duration (4.74±0.38 ms in CEM treated and 4.38±0.06 ms in WMTA treated group, P<0.001; (Figure 3C). The amplitude of AP was also affected by both application of CEM and WMTA in which they significantly decreased the amplitude of APs (81.57±2.75 mV, P<0.05 and 81.92±1.05 mV, P<0.01), but the suppressive effect of WMTA on the AP amplitude was more effective (Figure 3D). The AHP following APs which is an important determinant of neuronal firing was altered by exposure to either CEM or WMTA. The AHP amplitude was significantly (P<0.001) increased following extracellular perfusion of Ringer containing CEM (-14.77±0.61 mV) and WMTA (-16.63±1.12 mV), although the potentiating effect of WMTA was more effective (Figure 3E). The area under the curve for the AHP was also significantly increased (Figure 3F) when cells were exposed to either CEM (-2.48±0.18 mV, P<0.01) or (-4.01±0.48 mVs, P<0.001), although the effect of WMTA was significantly more effective than CEM (P<0.01). Application of phosphate solution as a vehicle of CEM did not significantly affect the neuronal excitability (data not shown).

The effects of CEM and WMTA on the AHP amplitude was independently evaluated with lower Ca^2+^ Ringer solution (i.e. extracellular concentration of Ca^2+^ was reduced to half) in a separate experiment. Under this condition, a significant reduction in the amplitude of AHP was observed (-10±0.2 mV, n=10; Student-t test, P<0.01) when compared to the value recorded in the normal Ringer concentration (-11.17±0.66 mV, n=36). By contrast, upon addition of either CEM or WMTA to the low Ca^2+^ Ringer, a significant enhancement in the AHP amplitude of F_1_ neurons was observed (Figure 4). However, the enhancing effect of WMTA on AHP amplitude was significantly more effective than CEM (P<0.001, n=8 in each treated group).

## Discussion

In the present study, we aimed to investigate whether neuronal electrophysiological characteristics including excitability and action potential configuration can be affected by direct application of WMTA and CEM, as dental biomaterials, using intracellular recording. Findings showed that both WMTA and CEM reduced the cell excitability and altered the action potential characteristics, although WMTA was more effective than CEM. Invertebrate neurons offer many experimental advantages and actually complement vertebrate studies. They have proved to be useful for understanding of some physiological processes in the central nervous system. There are remarkable similarities between the molecular architectures of vertebrate and invertebrate systems. These similarities include neurotransmitters, receptors and signal transduction mechanisms and even conserved neural ion channels. Neural system of invertebrates including molluscs play a pivotal role in toxicity and efficacy testing of new pharmaceuticals and material, because of their lower metabolic rate and greater resistance to hypoxia, their exceptional size, accessibility to experimentation and robustness of some neurons and axons [28][29]. More recently, we reported evidence that WMTA not only possess anti-nociceptive effect on both formalin-induced neurogenic and inflammatory pains, but also prevents formalin-induced pain in rat orofacial formalin test [27]. These results suggest that MTA induces analgesic effect possibly by suppression the nerve excitability. There is behavioral and pharmacological evidence indicating that a similar modulatory system is involved in the nociceptive responses of rodents and snails [30]. The FMRF amide-related family of neuropeptides, which were shown to be involved in the modulation of nociceptive behaviors both in molluscs and mammals [30][31], exhibits an inhibitory effect on sensory neurons such as F_1_ neurons through activation of K^+^ channels including Ca^2+^dependent K^+^ channels [32][33]. These outward K^+^ channels which are known to be activated by an influx of extracellular Ca^2+^ through voltage-dependent Ca^2+^ channels [34][35] are responsible for the AHP in many neurons, including snail neurons [33][35]. The amplitude and duration of the AHP that follows APs have been previously shown to be the key determinant of neuronal excitability [36][37].

In the present study, the induced changes in excitability could be due to enhancement of outward potassium current. This hypothesis is supported by a significant increase in the amplitude of AHP which occurred following application of either WMTA or CEM. AHPs following an AP plays an important role in repolarizing the AP and in shaping the discharge properties, including firing frequency and pattern [38][39][40][41]. In many neurons, APs are followed by a rise in intracellular free Ca^2+^ concentration leading to a prolonged AHP. Takita et al. [42] reported that MTA significantly can stimulate proliferation of cultured human pulp cells. The ability of MTA to induce proliferation in human pulp cells was attributed to release of calcium from MTA into the culture media. It has also been proposed that Ca^2+^ release from MTA is responsible for bone morphogenetic protein-2 expression and calcification in human periodontal ligament cells [43]. Based on above mentioned reports and knowing the composition of WMTA and CEM, it is possible that Ca^2+^ released from these two calcium enriched biomaterials causes an increase in the Ca^2+^ entry, which in turn activates Ca^2+^ dependent K^+^ (KCa) outward currents underlying AHP in F_1_ neurons. To test whether release of Ca^2+^ from WMTA or CEM contributed to the enhancement of AHP amplitude observed in the presence of both dental materials, intracellular recordings were performed in low Ca^2+^ concentration Ringer before and after either WMTA or CEM application. The present results showed that both compounds have enhancing effect on AHP amplitude, suggesting the possible involvement of Ca^2+^ release from the applied dental materials. However, the ability of WMTA to release Ca^2+^ appears to be greater than CEM, possibly due to their different chemical compositions.

Electron probe microanalysis results revealed that CEM cement is mainly composed of CaO, P2O5, SO3, and SiO2 [5], but WMTA is primarily composed of tricalcium silicate and bismuth oxide.

A second possible explanation for the increase in the AHP amplitude and thereby decrease in neuronal excitability is that both WMTA and CEM cause an extracellular alkaline shift [5][44][45], which in turn can modulate voltage-gated Ca2+ channels function. Several studies reported that extracellular alkaline pH causes activation of voltage sensitive Ca^2+^ channels in excitable cells [46][47][48], which may ultimately induce K^+^ outward currents by activation of KCa channels and thereby decrease the neuronal excitability. However, this hypothesis needs to be evaluated on calcium spikes.

The results of this study also showed that treatment with WMTA and CEM resulted in a significantly shortened AP duration, although WMTA induced more suppressive effects. Several outward K^+^ channels currents play critical roles in determining AP repolarization, duration and frequency [37][49][50]. Therefore, this also confirms the contribution of outward K^+^ current in WMTA or CEM mediated shortening of action potential and lowering the firing frequency.

## Conclusions

For the first time electro-physiological evidence was provided demonstrating the suppressive cellular effects of WMTA and CEM on neuronal excitability. Both dental biomaterials reduced the neuronal activity possibly through enhancement of K^+^ outward current. Application of both dental materials shortened the AP, reduced the firing frequency and enhanced the AHP amplitude. This may possibly have analgesic and regenerative effects, though further research is needed for such a conclusion.
